# Effect of 4-weeks exercise program using wearable hip-assist robot (EX1) in older adults: one group pre- and post- test

**DOI:** 10.1186/s12877-023-04423-x

**Published:** 2023-11-08

**Authors:** Jang-hoon Shin, Naeun Byeon, Heeju Yu, Geonhyang Yun, Hyunjin Kim, Seungyeop Lim, Dongwoo Kim, Hwang-Jae Lee, Wan-hee Lee

**Affiliations:** 1 Department of Physical Therapy, Sahmyook University College of Health Science, Seoul, 01795 Republic of Korea; 2 Samsung Noble County, Yongin, 17099 Republic of Korea; 3 Robot Business Team, Samsung Electronics, Suwon, 16677 Republic of Korea

**Keywords:** Aged, Exercise, Robotics, Wearable electronic devices

## Abstract

**Background:**

Older adults have muscle loss and are at risk of falling. Recently, research in the healthcare field has been actively conducted, and Samsung Electronics has developed EX1, a hip joint assisted robot for exercise. This study aimed to verify the effect of a 4-week combined exercise program applying EX1 on older adults.

**Methods:**

This study design was an evaluator-blinded, pre- and post-test. A total of 21 older adults performed an exercise program consisting of walking and fitness wearing EX1 for 50 min per session, 3 days a week during the 4-week exercise period. For comparison before and after participating in the exercise program, the spatio-temporal parameters, pelvic movement were analyzed by G-Walk, functional outcomes were evaluated by TUG, muscle power were evaluated by RUSI, and waist-hip ratio were analyzed by Inbody. All data were analyzed before and after exercise using paired t-test, and the statistical significance level was set at 0.05.

**Results:**

In spatio-temporal parameters, stride length showed statistically significant improvements after exercise with EX1 (*P* < 0.01). Also, propulsion showed statistically significant improvements after exercise with EX1 (*P* < 0.01) Regarding changes of the gait posture, there was a statistically significant improvement in pelvic movement (*P* < 0.05). In the functional evaluation, the time required was statistically significantly reduced in the timed up and go test (*P* < 0.05).

**Conclusion:**

These results demonstrate that a 4-week exercise program with EX1 was effective in improving the functional gait of the elderly. However, because the participants were 21, it is difficult to generalize the results.

**Trial registration:**

Clinical Research Information Service, KCT0007367. Registered 08/06/2022.

## Background

As people age, their activity levels tend to reduce [1]. Falling is one of the most important factors for the elderly [2]. Exercise has been reported to have positive physical, psychological and social impacts [3]. It also provides emotional well-being, and such appropriate physical activity has a positive effect on the quality of life of an individual by helping with physical and mental health [4]. Previous study showed that water therapy and jogging exercises leads to improve lower extremities of elderly preventing the falling [5]. Another study showed that aquatic exercise can lead to improve gait and balance of older adults [2]. Aerobic exercise has an important effect on improving the overall health of the elderly, positively affecting changes in the physical function, and reducing abdominal fat [6]. It is effective in improving the risk factors for cardiovascular diseases such as blood pressure control [7]. Exercise has been shown to help prevent falls, a severe traumatic event that is common in old age [8]. However, Previous study showed that there is still little literature about the impacts of ultra-endurance practice on the aging process [9].

Aerobic exercises include walking, running, and jumping rope performed at various speeds and directions, and the exercise intensity and duration can be gradually increased according to the subject’s ability, such that it is more effective. Considering the relationship between the type of physical activity and the amount of energy consumption, walking, which can be performed anytime, anywhere, is not lower than other activities [10, 11]. Aging is closely related to the loss of muscle mass, and a significant reduction occurs, especially in the lower limbs, which can lead to physical dysfunction. Previous studies have reported that an effective resistance exercise program for the elderly improves physical function [12]. Since balance exercise is highly related to risk, it is an essential element in an exercise program [13]. Older adults are most concerned about falls, and balancing exercises are helpful in preventing falls. According to previous studies, the fall rate decrease after balance exercise in older adults [14]. Also, previous study showed that cognitive training for elderly can affect to heartrate positively. Benefits of yoga aren’t not specific to maintaining physical health.

In the field of healthcare, robot technology is being actively developed in the field of surgery and rehabilitation and is being used in clinical practice. Recently, beyond the use of robots limited to patients in the medical environment, the technological development of ‘daily assistance robots’ has been actively conducted as a concept of health management and assistance in daily life [15]. In particular, the walking aid robot has been developed into a more simplified form, minimized in weight, and worn like clothes, making it lightweight [16]. According to the previous research results using the walking aid robot, Gait Enhancing and Motivating System-Hip (GEMS-H, Samsung Electronics Co., Ltd., Korea), in order to enhancing the walking ability of older adults, it was shown that the walking speed and excessive muscle activity were reduced significantly when walking while wearing the robot, and a significant decrease in respiratory metabolic energy cost was observed [15]. The results of this prior study have the potential value of showing positive effects when exercising while wearing the robot. Recently, the Samsung Advanced Institute of Technology developed an exercise assistance robot named EX1 (Samsung Electronics Co., Ltd., Korea) in response to the widening of healthcare and the need for exercise for older adults [17]. This robot is a type of robot worn on the hip joint, has 2 thigh frames and 2 thigh straps for fixing, central processor and battery, inertial measurement unit, and two actuators which are located on the greater trochanters. It is very lightweight and customized to the wearer. It is equipped with an assistive mode which assists the wearer in exercising, such that they do not use too much force, and a resistive mode, which allows more force to be used by providing resistance during exercise. Using these functions, it is expected that it will be possible to combine various exercises, such as lower-extremity resistance exercise and aerobic exercise, which are necessary for the elderly. Therefore, the purpose of this study is to evaluate the effectiveness of using EX1 for performing exercise programs that combine strength exercise, balance exercise, and walking exercise which have been widely used in older adults in the past.

## Methods

### Study design and setting

This research was planned as an interventional study that was conducted at a single center. The study was carried out following the principles of good clinical practice. The study was approved by the Institutional Review Board of Sahmyook University (IRB No. SYU 2200–04-015–001). Informed consent was obtained from all participants prior to the study. This study was structured according to the CONSORT reporting guidelines [18].

### Participants

Older adults aged ≥ 65 years without a history of central nervous system diseases from a silver town in Yongin-si, Gyeonggi-do, Korea were included in the study. The exclusion criteria were as follows: inability to walk independently due to visual field defects or fractures; musculoskeletal disorders; inability to understand and participate in an exercise program due to severe cognitive decline; inability to exercise due to uncontrolled high blood pressure and diabetes; individuals who are at risk of falling while walking due to severe dizziness; and auditory dysfunction or defects. Elderly people who had no problems walking independently in the laboratory and whose postural alignment was visually correct and had no functional impairment due to postural alignment. The exclusion criteria for EX1 were as follows: individuals of height that did not fit the size of EX1 and individuals who were highly obese with a body mass index (BMI) of 35 or higher. Based on the selection criteria, a total of 24 older adults were recruited, three participants dropped out, and the final 21 participants (women, *n* = 17; men, *n* = 4) were included.

All the eligible participants were given a detailed explanation of the purpose and required information regarding the study by the researcher and information that they could withdraw from the study at any time. All the participants signed a consent form. General characteristics were investigated using self-reported questionnaires. We calculated that we would need 20 evaluable patients under the assumption of a two-sided type I error of 5% and a power of 80% (t-test). Rounding up and accounting for an expected loss to follow-up of 5% (in terms of missing primary outcome data) implied that we would require a sample size of ~ 24 patients. Our final sample size was about 20% larger than this computed requisite sample size.


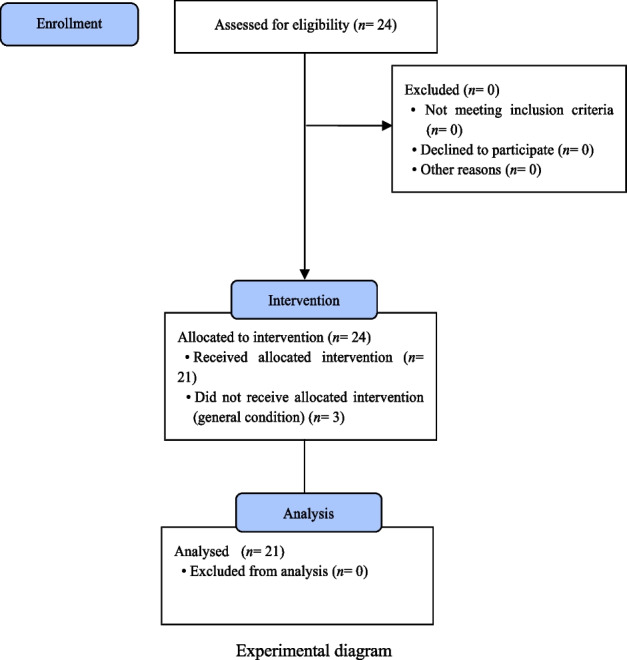


### EX1

#### Hardware of EX1

EX1 is an exoskeleton robot that generates assistive and resistive torques for hip flexion and extension. It is worn on the user’s waist and thighs and is lightweight, weighing approximately 2.9 kg. EX1 consists of two actuators, a control pack, power switch, rechargeable battery, thigh support frame, thigh support strap and a waist belt. Each actuator consists of motor, angular position sensor and controller. The Control pack consists of central processor, inertial measurement unit (IMU) sensor and a rechargeable battery. Actuators create assistive and resistive torques and provide them to the user’s thigh through the thigh support frame. The user adjusts the torque through a Bluetooth connection to the mobile device. It can be used for a period of 2 h while walking continuously at a speed of 4 km/h, and the maximum noise level is less than 45 dB at the height of user’s ear.

#### Software of EX1

EX1 is operated by the delayed output feedback control (DOFC) method for generating torque, which does not include the gait phase estimator or reference lookup. First, when the user voluntarily flexes or extended the hip joint while wearing EX1, sensors located on both sides of the hip joint of EX1 sense it at 100 Hz. After the information is collected, it is smoothed using a low-pass filter; subsequently, an appropriate assistive or resistive torque is calculated in a central processor. The actuator then generates an assistive or resistive force at the hip joint of the user. The left and right actuators alternately generated forces in opposite directions (hip flexion and hip extension) and the torque increased proportionally as the user’s walking speed increased.

### Intervention

The combined exercise program was designed to improve physical function and strength of the lower extremities in older adults. The exercise consisted of 5 min of warm-up, 15 min of strength exercise, 5 min of rest, 20 min of aerobic exercise, and 5 min of cool-down. It took 50 min in total. The combined exercise program was conducted three times a week for four weeks, for a total of 12 sessions. All subjects performed a complex exercise program after wearing EX1.

The strength exercises included kick back, knee- ups, squats, and reverse lunges for 15 min. The exercise methods used are listed in Table 1. After strength exercise, the subjects sat on a chair and took a break for 5 min. For aerobic exercise, after walking on an indoor flat surface for 10 min, the participants climbed three-story stairs. After that, they walked on flat ground again for 10 min and climbed the 3rd floor stairs once more, for a total of 20 min. The stairs were 27 cm wide and 15 cm high, with 24 steps on each floor.

**Table 1:** Strength exercise composition

Exercise	Exercise name	Composition	Hour
Strength exercise	Kick back	After maintaining the correct standing posture, hold on to the handrail to prevent falls. After shifting the weight to the left leg, straighten the knee of the right leg and extend the hip joint back. After 12 repetitions of the right leg, do the same with the left leg. Perform a total of 4 sets	4 min
	Knee up	After maintaining the correct standing posture, shift the weight to the left side. Bend the right leg 90 degrees so that the knee is higher than the waist. Alternate right and left sides 24 times. A total of 5 sets were conducted	4 min
	Squat	Spread your feet shoulder-width apart. Distribute the center of gravity evenly on both feet. Push your hips back as if you were sitting in a chair and bend your knees. Do this 10 times for a total of 3 sets	3 min
	Reverse lunge	After maintaining the correct standing posture, shift the weight to the left leg. Take a big step with your right leg back. Bend your left leg and lower your right knee until it touches the floor. After performing 10 times with the left leg, do the same with the right leg for a total of 4 sets	4 min

As for the intensity of exercise, a gradual overload was applied each week using the mode and intensity of EX1 to improve the muscle strength. The same intervention was applied to people, and as an exception, elderly people whose physical condition deteriorated that day were allowed to participate in exercise at an adjusted intensity. All the exercises were performed under the supervision of a physical therapist, sufficient rest was provided according to the subject’s condition.

### Outcome measurements

#### Primary outcome

The gait data were collected during the performing 10-m walk test (10MWT) and timed up and go test (TUG) using a wearable IMU (BTS G-Sensor 2, BTS Bioengineering, Korea). It has shown that the IMU device has excellent reliability for measuring 10MWT and TUG with intraclass correlation coefficient 0.728 – 0.969 for the 95% confidence interval [19, 20]. This wearable IMU was placed at the sacral vertebrae at the S1–S2 level for the 10MWT and at the lumbar vertebrae as the L2 level for the TUG embedded into an ergonomic waist belt [21]. The device was connected to a notebook computer using Bluetooth. The sample recording frequency was 100 Hz. The data were collected using the software G-Studio (BTS Bioengineering).

- *10MWT*: The participants walked along a linear pathway of 10 m from a starting point to a stop line at a comfortable pace. Stopwatch recording was started when the toe passes at the 2 m point of the acceleration phases and stopped when the toe passed at the 8 m point of the deceleration phases. The gait speed was measured in a constant velocity phase of 6 m with a stopwatch [22]. The study gathered information on the stance phase ratio, stride length propulsion, and range of movement of the pelvis using IMU. Two trials were recorded and used for data analysis.
- *TUG*: The participants were asked to sit on a chair without armrests. At the “start” signal, they were requested to rise from a chair, walk at their usual pace a distance of 3 m, make a 180° turn around a cone, walk back to the chair, and performed a second 180° turn to sit down and end the test [23]. In this measurement, we acquired functional evaluation as follows: TUG time duration, sit to stand trunk extension range, stand to trunk flexion range. Three trials were performed and used for data analysis.

#### Secondary outcome

##### One-leg standing test (OLST)

The static balance was measured using the OLST. The participants were asked to maintain a one-legged stance for as long as possible during their eyes open test and were allowed to cross their arms over the chest [24]. An assessor measured the amount of time that the participants were able to stand on their dominant leg using a stop-watch with the maximum timed set at 60 s [25]. The participants were timed as soon as their foot left the floor and stopped once the foot touched the ground, the foot moved on the floor, the legs touched each other, an arm was uncrossed, or the maximum allotted time was reached [26]. Three trials were recorded, and the average scores from each trial were used for the data analysis.

##### Surface Electromyography (sEMG)

sEMG (BTS Bioengineering, Milano, Italy) was used to examine the effect on muscle activity. The data were sampled at 1000 Hz, amplified 1000 times, relayed to a computer-based data acquisition and analysis system Muscle activation data were obtained by using analysis software EMG Analyzer v2.9.37.0 (BTS Bioengineering, Milano, Italy). The collected signal was subjected to a bandpass filter at 20—450 Hz and the root mean square (RMS) was processed with a 50 ms mobile window. The electrode sites were prepared by shaving and cleaning the area with alcohol in advance. Subsequently, the surface EMG was attached according to the Surface Electromyomraphy for the Non-Invasive Assessment of Muscles guidelines. The electrodes were oriented parallel to the fiber direction of the rectus abdominis (RA) and biceps femoris (BF) muscles. Maximal voluntary contraction (MVC) was used for the normalization of surface EMG signals. The MVC test was performed based on the manual muscle test method for each muscle. After sufficient rest, the same evaluation was conducted once more. The the RMS value of the middle 3 s were calculated, excluding the first 1 s and last 1 s. Based on the maximum contraction value, the activity value of each muscle was normalized (%). The number of measurements was twice for each test. After measuring the MVC, the subjects walked 10 m at a comfortable pace twice. By attaching an inertial sensor to the L5 level, it automatically distinguished between the stance and swing phases during walking. The average muscle activity of the BF and RA was divided for each phase and analyzed by integrating it into ensemble graph. The muscle activity for each phase was expressed based on the MVC value of 100%.

##### Rehabilitative Ultrasound Imaging (RUSI)

The thicknesses of the RA and quadriceps muscles were measured using a real-time brightness mode ultrasound scanner (SONON 300, Healcerion, Seoul, Korea). The examiner measured twice when the subject was comfortably relaxed and twice in the state of MVC.

- *RA*: The image of the RA gain was set at 76 dB in the convex array. The RA was measured on the dominant side by a skilled examiner. With the participants in the hook-lying position with their knee flexed, measurements were taken at rest. The transducer was placed 2 cm above the umbilicus from the midline. When measuring the MVC, the subjects placed their hands behind their heads, flexed their trunk, and contracted the RA muscle as much as possible and hold for 3 s. The thickness of the RA was measured vertically at the mid-point of the width of the belly between the inside edges of the superior and inferior fascial borders.
- *Quadriceps muscle*: The image of quadriceps muscle gain was set at 64 dB in a linear array. The quadriceps muscle was measured in the dominant leg by a skilled examiner. The subjects lay supine on the bed with their legs fully extended and muscles relaxed. The transducer was placed 15 cm above the top of the subject’s patella. When measuring the MVC, the subject sat on the bed, pressed a towel under the knee, and contracted the thigh as much as possible [27], and hold for 3 s. The quadriceps muscle thickness was obtained by calculating the distance between the cortex of the femur and the most superficial fascia [28]. The following formula was used to obtain the contraction ratio:

$$\mathit'\mathit(thickness\mathit\;at\mathit\;contraction\mathit\;\mathit-\mathit\;thickness\mathit\;at\mathit\;rest\mathit)\mathit\;\mathit/\mathit\;thickness\mathit\;at\mathit\;rest\mathit\;\mathit\ast\mathit\;\mathit{100}\mathit\;\mathit(\mathit\%\mathit)\mathit'$$

##### Waist-hip ratio (WHR)

The WHR was measured using an Inbody 770 (Biospace Inc., Seoul, Korea). The participants took the electrodes in their hands, with the angle of the hands to the body at approximately 30°. Subsequently, the participant pushed the buttons on the handles until the measurement was finished, while maintaining a fixed and immobile posture for the recording [29]. The Inbody measurements were recorded once and were used for data analysis.

### Statistical analysis

PAWS statistics 18 (SPSS Inc., Quarry Bay, Hong Kong) was used for all the statistical analyses. The Shapiro–Wilk test was performed to confirm the normality of the participant characteristics. All data have been expressed as mean (SD; standard deviation). A paired t-test was used to compare the outcome between the pre- and post- exercise programs. The statistical significance threshold was set at *P* < 0.05.

## Results

### Baseline participant characteristic

The baseline characteristics of the 21 older adults are summarized in Table 2.

**Table 2:** Characteristics of participants (*N* = 21)

**Sex (male / female)**	4 / 17
**Age (year)**	76.38 (4.97)
**Height (cm)**	158.69 (8.18)
**Weight (kg)**	60.06 (8.71)
**BMI (kg/m**^**2**^**)**	23.81 (2.34)

Values are presented as number or mean (SD)

*BMI* Body Mass Index

### Primary outcome

**Fig. 1:**
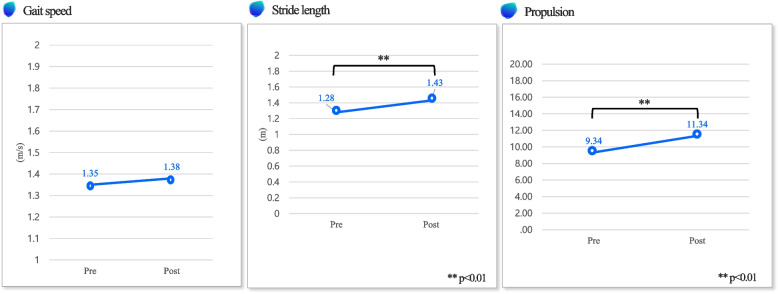
Spatio-temporal parameters—this figure shows gait speed, stride length, and propulsion change between pre- and post- exercise program

**Fig. 2:**
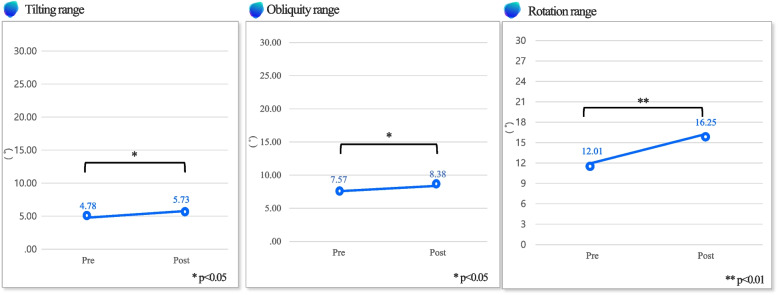
Pelvic movement range – this figure shows tilting range, obliquity range, and rotation range change between pre- and post- exercise program

Table 3 describes the outcomes of the spatio-temporal parameters. The stride length increased significantly after exercise (*P* < 0.01). The propulsion also increased statistically significantly after exercise than before (*P* < 0.01) (Fig. 1). Table 3 shows the pelvic movement range outcomes. There were significant increases in the pelvic movements, tilting range (*P* < 0.05), obliquity range (*P* < 0.05) and rotation range (*P* < 0.01) between pre- and post-intervention (Fig. 2).

**Table 3:** Primary outcome measurements (*N* = 21)

Variable	Pre	Post
**Spatio-temporal parameters**		
Gait speed (m/s)	1.35 (0.15)	1.38 (0.18)
Stance phase ratio (%)	59.29	59.89
Stride length (m)^**^	1.28 (0.18)	1.44 (0.19)
Propulsion^**^	8.9 (2.41)	10.8 (2.48)
**Pelvic movement range**		
Tilting range (˚)^*^	4.78 (1.49)	5.73 (2)
Obliquity range (˚)^*^	7.57 (2.89)	8.38 (2.84)
Rotation range (˚)^**^	12.01 (4.35)	16.25 (5.84)

Values are presented as mean (SD)

^**^P < 0.01

^*^P < 0.05

### Secondary outcome

**Fig. 3:**
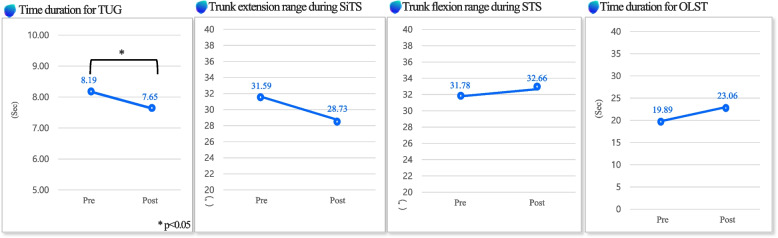
Functional evaluation—this figure shows time duration for timed up and go test, trunk extension range during sit to stand, trunk flexion range during stand to sit, and time duration for one-leg standing test change between pre- and post- exercise program

**Fig. 4:**
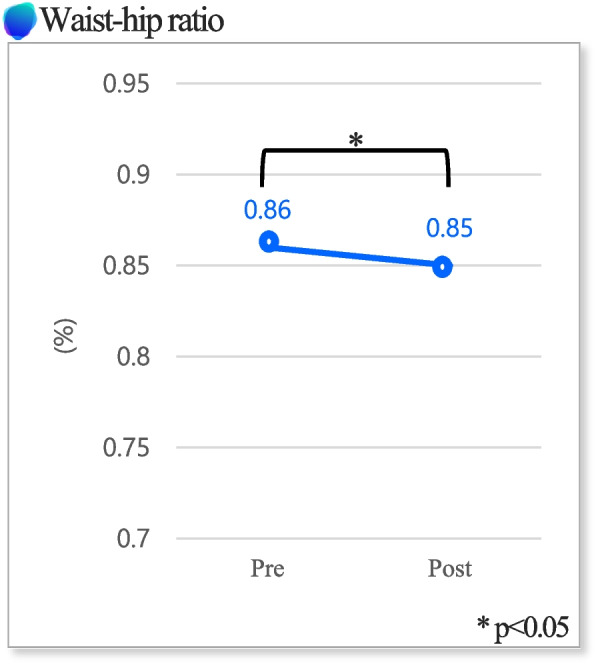
Waist-hip ratio – this figure shows waist-hip ratio change between pre- and post- exercise program

Table 4 describes the functional evaluation outcomes, power outcomes, and the WHR outcomes. There was a statistically significant difference in the TUG time duration which reduced after exercise (*P* < 0.05). (Fig. 3). In addition, there was a statistically significant difference in the WHR which also reduced after exercise (*P* < 0.05) (Fig. 4).

**Table 4:** Secondary outcome measurements (*N* = 21)

Variable	Pre	Post
**Functional evaluation**		
TUG TD (sec)^*^	8.19 (1.23)	7.65 (1.25)
SiTS Trunk E R (˚)	31.59 (7.45)	28.73 (6.07)
STS Trunk F R (˚)	31.78 (9.72)	32.66 (14.13)
OLST TD (sec)	19.89 (19.2)	23.06 (18.96)
**Muscle power**		
RA (during stance phase) (*µ*V)	10.24 (12.15)	11.63 (9.25)
BF (during swing phase) (*µ*V)	38.04 (27.38)	41.55 (17)
RA contraction ratio (%)	206 (59)	217 (46)
Quadriceps thickness (mm)	16.07 (3.56)	18.13 (3.81)
**Waist-Hip Ratio**		
WHR (%)^*^	0.86 (0.06)	0.85 (0.07)

Values are presented as mean (SD)

*TUG TD* Timed up and Go test Time duration, *SiTS Trunk E R* Sit to Stand Trunk Extension Range, *STS* Stand to Sit Trunk Flexion Range, *OLST TD* One Leg Stance Test Time Duration, *RA* Rectus Abdominus, *BF* Biceps Femoris, *WHR* Waist-Hip Ratio

^*^P < 0.05

## Discussion

This study aimed to evaluate the effectiveness of a 4-week exercise program using EX1 in older adults. The composition of the exercise program was created by combining strength and aerobic exercise in consideration of the mechanical characteristics of EX1 and the physical characteristics of older adults.

During the 10-m walking test, the speed increased by 1.93% during comfortable walking, although it was not significant when comparing before and after four weeks. In particular, the speed increased the most at the mid-point which is the results of assistive mode of the EX1 exercise. In the assistive mode, the leg was lifted high to increase the hip flexion angle, which increased the stride length, and affected the speed of straight walking simply by trying to move forward. Therefore, it suggests that a simple straight walking speed increase is possible only with assistive mode-applied exercise. As a result, after 4 weeks of exercise, the straight walking speed increased, which was due to the increase in the other variables (increase in the stride length, pelvic angle, etc.); a healthy person reaches 60% of the stance phase and 40% of the swing phase in the gait cycle [30]. The older adults who participated in this study confirmed that the ratio of the phases reached the optimal ratio after exercise. In addition, the ratio of the swing phase increased when the assistive mode was used for 2 weeks in the first half, and the ratio of the stance phase increased when the resistive mode was used in the second half. This is because the assistive mode increases the single-leg support time by lengthening the swing time, and the resistance works in the opposite direction in the resistive mode. As the exercise progressed, the stride length increased significantly by 12.42% (*P* < 0.01). This suggests that exercise using EX1 improved the walking ability of subjects to move a longer distance with a wider stride. The propulsion showed a significant increase of 21.29% after exercise (*P* < 0.01). This was judged to be the effect of robot-assisted exercise, which helps continuous forward walking by assisting hip joint flexion. As EX1 assisted in the motion of lifting the thigh, the subject was able to flex the hip joint easily and rapidly. Additionally, while the hip joint was flexed higher, the center of the body moved in the direction of walking, and the force pushing the ground increased.

During walking, the pelvis is observed to move in three planes: tilt movement that can be seen in the sagittal plane, obliquity movement that can be seen on the frontal plane, rotation movement that can be observed on the transverse plane [31]. The movement of the pelvis during walking reduces movement of the center of mass in the vertical and horizontal directions. Therefore, it reduces energy consumption and makes walking economical and efficient. The range of pelvic movement increased as walking speed increased. In this study, the pelvic angle range during walking increased significantly in all three planes. The increase in the rotation range of the pelvis was 35.33%. During walking, the pelvis generally moved between 3 ˚ and 14 ˚ in the transverse plane. In this study, the pelvis was rotated from 12.01 ˚ to 16.25 ˚ while walking. According to previous studies, this phenomenon occurs when pedestrians walk at their preferred speeds [32]. In this study, the results were extracted by walking at the speed preferred by the subjects. This is because the increase in the pelvic rotation range during walking enables walking with the feet extended farther, and the center of mass of the body lowered, leading to stable walking. During walking, the pelvis typically moves between 2 ˚ and 5 ˚ in the sagittal plane. In this study, the pelvis was rotated from 4.63 ˚ to 5.73 ˚ increasing 19.82%. Therefore, it can be seen that the pelvic tilt is gradually increasing along the normal range. During walking, the pelvis generally moves between 6 and 11 degrees from the frontal plane. In this study, the pelvis moved up to 7 ˚ – 8 ˚ increasing 10.69% [32]. Therefore, it can be seen that the pelvic obliquity is gradually increasing along the normal range. Overall, the range of movement of the pelvis in three aspects increased while adhering to the normal range. In addition, the internal abdominal oblique and external abdominal oblique muscles are involved in the rotation range of the pelvis. Through the results of this study, it can be seen that EX1 contributed to the activation of the subject’s core muscle.

In general, TUG is recognized as having a high risk of falling if it takes more than 14 s [33]. The time required for the TUG test decreased significantly by 6.63%. This result suggests that the EX1 4-week exercise program has positive effects in dynamic balance abilities and gait speed in older adults. When standing up, the extension range was observed to slightly decreased. It is presumed that this is because the subjects quickly switched to walking without fully extending their trunk after standing. The increase in the range of motion of the trunk in the stand-to-sit motion serves to increase stability in terms of falls by sufficiently bending the trunk when older adults sit on a chair. Older adults have changes in their gait pattern due to various geriatric diseases [34]. In addition, the extension angle of the hip joint decreases, the anterior pelvic tilt increases, the range of motion of various joints, and the range of motion of the trunk also tends to decrease showing a ‘cautious gait pattern’ [35]. However, as a result of the 4-week exercise program applied with EX1, it was confirmed that there was an improvement in the range of motion of the trunk. This is judged to be because it contributed to the positive improvement of factors related to the dynamic balance ability, such as increasing the range of motion of the pelvis, muscle strength, and widening the stride length. It is judged that the range of motion when sitting down has improved, and an increase in the range of motion of the trunk reduces energy consumption and enables efficient execution of these motions. The average OLST endurance time increased after exercise program. Older adults use the hip strategy, at the highest rate among fall prevention strategies [36], and it has been shown that the hip joint-centered muscle strength exercise of EX1 is positively helpful to the hip strategy. Moreover, in order to keep one leg lifted, an appropriate contraction ratio of the hip abductor and adductor muscles is required [37]. Exercise using EX1 is considered to have a positive effect on the efficient contraction of the hip abductor and hip adductor muscles.

The muscle activity of the RA in the stance phase during walking showed a tendency to increase by 13.65% to maintain the neutral position of the trunk in a dynamic state with EX1 worn on the waist. In the swing phase during walking, the muscle activity of BF was not significant, but increased by 9.23%. Because the BF is an important muscle for deceleration control at the end of the swing phase, it can be seen that the EX1 exercise helps not only acceleration but also deceleration control, contributing positively to maintaining stable gait in the elderly [38]. Since the driving axis of EX1 is centered on the hip joint, the muscle strength of the quadriceps muscle, which directly resists the motion of hip joint flexion, increases, and the RA muscle increases to maintain neutrality of the trunk.

The WHR decreased significantly by 1.71% after the 4-week EX1 exercise program (*P* < 0.05). In this study, a combined exercise program of aerobic exercise and resistance exercise was implemented considering the characteristics of EX1’s assistive mode and resistive mode for 4 weeks; the increase in physical activity increased the active calorie of the subjects and repeated hip joint exercises showed this result. However, the abdominal fat rate was very closely related to the daily intake [39]; however, limitations followed because the subject’s food intake was not controlled.

### Limitations

This study has several limitations. Because there was no control group, it is difficult to confirm whether the results of this study are effect of EX1. In addition, because the follow up was not performed, it is difficult to confirm the residual effects of the exercise. Since the subjects who participated in the study were older adults, the exercise period of 4 weeks was rather short to improve the muscle strength. In the future, it will be necessary to design a long-term exercise program effect for simple exercise such as walking using EX1 for older adults. However, this study is valuable because it is one of the few studies that investigated the effects of long-term exercise using a wearable robot. In addition, it is a trendy study that combines exercise methods that fit the recent trend of fitness and walking with a robot.

## Conclusions

The exercise program applied with EX1 showed a positive effect on the improvement of function in older adults. Although positive effects on muscle strength improvement were not achieved, gait function and balance ability were shown to be effective. Therefore, the 4-week combined exercise program applied with EX1 was effective in improving the function of older adults.

## Data Availability

The datasets used and/or analyzed during the current study are available from the corresponding author on reasonable request.

## Abbreviations

GEMS-H: Gait Enhancing and Motivating System-Hip
BMI: Body mass index
IMU: Inertial measurement unit
DOFC: Delayed output feedback control
10MWT: 10-Meter walk test
TUG: Timed up and go test
OLST: One-leg standing test
sEMG: Surface Electromyography
RMS: Root mean square
RA: Rectus abdominis
BF: Biceps femoris
MVC: Maximal voluntary contraction
RUSI: Rehabilitative Ultrasound Imaging
WHR: Waist-hip ratio
SD: Standard deviation

## References

[1] Burton E, Farrier K, Galvin R (2019). Physical activity programs for older people in the community receiving home care services: systematic review and meta-analysis. Clin Interv Aging.

[2] Irandoust K, Taheri M, Mirmoezzi M (2019). The effect of aquatic exercise on postural mobility of healthy older adults with endomorphic somatotype. Int J Environ Res Public Health.

[3] Park JM, Han SH (2003). The effect of exercise program on health and depression in the elderly. J Korean Acad Nurs.

[4] Guyatt GH, Van Zanten SV, Feeny DH, Patrick DL (1989). Measuring quality of life in clinical trials: a taxonomy and review. CMAJ.

[5] Taheri M, Farzian S, Esmaeili A, Shabani E. The effect of water therapy and jogging exercises on the health-related factors of physical fitness of elderly women. Int J Sport Stud Health. 2023;3(2):e114813.

[6] Timmons JF, Minnock D, Hone M, Cogan KE, Murphy JC, Egan B (2018). Comparison of time-matched aerobic, resistance, or concurrent exercise training in older adults. Scand J Med Sci Sports.

[7] Bai X, Soh KG, Omar Dev RD, et al. Aerobic exercise combination intervention to improve physical performance among the elderly: a systematic review. Front Physiol. 2022;12:2311. 10.3389/fphys.2021.798068 PMC8764279 35058805

[8] Sherrington C, Michaleff ZA, Fairhall N (2017). Exercise to prevent falls in older adults: an updated systematic review and meta-analysis. Br J Sports Med.

[9] Vancini RL, dos Santos AM, de Lira CA, Theodoros Nikolaidis P, Knechtle B (2022). Is It Possible to Age Healthy by Performing Ultra-endurance Exercises?. Int J Sport Stud Health.

[10] Sung H (2009). Impacts of walking activity in daily life on individual health improvement. Korea Spatial Plan Rev.

[11] Vuori I (1998). Does physical activity enhance health?. Patient Educ Couns.

[12] Bårdstu HB, Andersen V, Fimland MS (2020). Effectiveness of a resistance training program on physical function, muscle strength, and body composition in community-dwelling older adults receiving home care: a cluster-randomized controlled trial. Eur Rev Aging Phys Act.

[13] Osoba MY, Rao AK, Agrawal SK, Lalwani AK (2019). Balance and gait in the elderly: a contemporary review. Laryngoscope Investig Otolaryngol.

[14] Lesinski M, Hortobágyi T, Muehlbauer T, Gollhofer A, Granacher U (2015). Effects of balance training on balance performance in healthy older adults: a systematic review and meta-analysis. Sports Med.

[15] Lee H-J, Lee S, Chang WH (2017). A wearable hip assist robot can improve gait function and cardiopulmonary metabolic efficiency in elderly adults. IEEE Trans Neural Syst Rehabil Eng.

[16] Shin JH, Park HK, Jung J (2022). A Study on Energy Efficiency in Walking and Stair Climbing for Elderly Wearing Complex Muscle Support System. Phys Ther Rehabil Sci.

[17] Lee S-H, Lee H-J, Kim K, Lee B-H, Kim Y-H (2022). Effect of exercise using an exoskeletal hip-assist robot on physical function and walking efficiency in older adults. J Pers Med.

[18] Schulz KF, Altman DG, Moher D (2010). CONSORT 2010 statement: updated guidelines for reporting parallel group randomised trials. J Pharmacol Pharmacother.

[19] Yazici G, Volkan M, Çobanoğlu G (2020). The reliability of a wearable movement analysis system (G-walk) on gait and jump assessment in healthy adults. J Exerc Ther Rehabil.

[20] Kleiner AFR, Pacifici I, Vagnini A (2018). Timed Up and Go evaluation with wearable devices: validation in Parkinson’s disease. J Bodyw Mov Ther.

[21] Buraschi R, Pollet J, Villafañe JH, Piovanelli B, Negrini S (2022). Temporal and kinematic analyses of timed up and go test in chronic low back pain patients. Gait Posture.

[22] Marques A, Cruz J, Quina S, Regêncio M, Jácome C (2016). Reliability, agreement and minimal detectable change of the timed up & go and the 10-meter walk tests in older patients with COPD. COPD..

[23] Mulas I, Putzu V, Asoni G, Viale D, Mameli I, Pau M (2021). C linical assessment of gait and functional mobility in Italian healthy and cognitively impaired older persons using wearable inertial sensors. Aging Clin Exp Res.

[24] Springer BA, Marin R, Cyhan T, Roberts H, Gill NW (2007). Normative values for the unipedal stance test with eyes open and closed. J Geriatr Phys Ther.

[25] Michikawa T, Nishiwaki Y, Takebayashi T, Toyama Y (2009). One-leg standing test for elderly populations. J Orthop Sci.

[26] Ortega-Pérez de Villar L, Martínez-Olmos FJ, Junqué-Jiménez A (2018). Test-retest reliability and minimal detectable change scores for the short physical performance battery, one-legged standing test and timed up and go test in patients undergoing hemodialysis. PloS one..

[27] Shin J-H, Lee W-H (2020). The effects of two different visual feedback exercise tools based on rehabilitative ultrasound imaging in the elderly. Phys Ther Rehabil Sci.

[28] Shin J, Lee W (2021). Muscle activity based on real-time visual feedback training methods by rehabilitative ultrasound image in elderly and relationship between heckmatt scale, muscle thickness and tone: a pilot study. Phys Ther Rehabil Sci.

[29] Pasdar Y, Moradi S, Abdollahzad H (2019). Accuracy of waist to hip ratio calculated by bioelectric impedance device in the Ravansar non-communicable disease cohort study. Top Clin Nutr.

[30] Iosa M, Fusco A, Marchetti F (2013). The golden ratio of gait harmony: repetitive proportions of repetitive gait phases. BioMed Res Int.

[31] Smith B, Ashton KM, Bohl D, Clark RC, Metheny JB, Klassen S (2006). Influence of carrying a backpack on pelvic tilt, rotation, and obliquity in female college students. Gait Posture.

[32] Lewis CL, Laudicina NM, Khuu A, Loverro KL (2017). The human pelvis: variation in structure and function during gait. Anat Rec.

[33] Arnold CM, Faulkner RA (2007). The history of falls and the association of the timed up and go test to falls and near-falls in older adults with hip osteoarthritis. BMC Geriatr.

[34] Alexander NB (1996). Gait disorders in older adults.

[35] Herman T, Giladi N, Gurevich T, Hausdorff J (2005). Gait instability and fractal dynamics of older adults with a “cautious” gait: why do certain older adults walk fearfully?. Gait Posture.

[36] Rubenstein LZ (2006). Falls in older people: epidemiology, risk factors and strategies for prevention. Age Ageing.

[37] Porto JM, Freire Junior RC, Bocarde L (2019). Contribution of hip abductor–adductor muscles on static and dynamic balance of community-dwelling older adults. Aging Clin Exp Res.

[38] Lockhart TE, Kim S (2006). Relationship between hamstring activation rate and heel contact velocity: factors influencing age-related slip-induced falls. Gait Posture.

[39] Chen W, Liu Y, Yang Q (2016). The effect of protein-enriched meal replacement on waist circumference reduction among overweight and obese Chinese with hyperlipidemia. J Am Coll Nutr.

